# Exploring Technology Acceptance of Healthcare Devices: The Moderating Role of Device Type and Generation

**DOI:** 10.3390/s24247921

**Published:** 2024-12-11

**Authors:** Seieun Kim, Yinai Zhong, Jue Wang, Hak-Seon Kim

**Affiliations:** 1 Department of Global Hospitality Management, Kyungsung University, Busan 48434, Republic of Korea; celinekim@ks.ac.kr (S.K.); yinai@ks.ac.kr (Y.Z.); 2 School of Global Studies, Kyungsung University, Busan 48434, Republic of Korea; 3 School of Hospitality & Tourism Management, Kyungsung University, Busan 48434, Republic of Korea

**Keywords:** Internet of Things (IoT), healthcare device, unified theory of acceptance and use of technology (UTAUT), attitude, trust, perceived risk, generation, device type

## Abstract

The increasing adoption of healthcare devices necessitates a deeper understanding of the factors that influence user acceptance in this rapidly evolving area. Therefore, this study examined the factors influencing the technology acceptance of healthcare devices, focusing on radar sensors and wearable devices. A total of 1158 valid responses were used to test hypotheses, mediation, and moderation effects using SmartPLS 4.0. The results highlighted the significant role of performance expectancy, effort expectancy, social influence, facilitating conditions, and perceived risk in shaping user attitudes and trust, which in turn influence behavioral intention. The findings suggested that attitudes fully mediate the effects of performance expectancy and effort expectancy on behavioral intention, while social influence, facilitating conditions, and perceived risk exhibit partial mediation. Moderation analysis revealed significant effects of generation on the relationship between attitude, trust, and behavioral intention. Additionally, device type moderated the effect of trust on behavioral intention, showing a different influence between radar sensors and wearable devices. These findings provide theoretical contributions by extending the unified theory of acceptance and use of technology (UTAUT) model and offering practical implications for manufacturers and policymakers to tailor strategies that foster positive attitudes, enhance trust, and address generational and device-specific differences in healthcare technology adoption.

## 1. Introduction

Information and communication technology (ICT) has transformed the healthcare industry [1]. The Internet of Things (IoT)—a system of interrelated computing devices and mechanical and digital machines with the ability to transfer data over a network without requiring human-to-human or human-to-computer interaction—is one major engine powering this change [2]. These typical characteristics enable the IoT to support decision-making processes, optimize resultant actions, and facilitate service attributes in a variety of situations, such as those related to healthcare service delivery [3]. The application of IoT in healthcare is expected to witness significant growth. The global IoT in healthcare market is expected to reach USD 83.81 billion by 2024, growing at a CAGR of 9.91% from 2024 to 2029, and will further peak at USD 134.4 billion by 2029 [4]. In the maturation of the integration of IoT with the activities of the healthcare sector, IoT can redefine healthcare services and their provision of monitoring and management, thus benefiting patients, healthcare providers, and society at large [5].

The IoT consists of four key layers: perception, network, middleware, and application. The perception layer performs the task of collecting data about the environment, such as vital signs in healthcare. The network layer processes the data transmission and delivery to relevant parties in real time. A middleware layer handles data management and integration, while the application layer supports user interaction for healthcare providers and patients in monitoring, analyzing, and decision-making using IoT [6]. This architecture is widely used in many applications across different industries, especially in healthcare where IoT devices play an important role in remote monitoring, early diagnosis, and personalized treatment plans [7].

With the advent of IoT solutions, the healthcare industry has made notable strides, particularly in home healthcare. Home IoT healthcare devices, including stationary radar sensors and wearable devices, enable continuous monitoring and management of health conditions by collecting critical data, such as heart rate, respiration, and glucose levels. This information can be transmitted to healthcare providers, enabling timely interventions when needed [8]. Recent advances in IoT have introduced sensor fusion, which integrates multiple sensor inputs to improve measurement accuracy in wearable devices. For example, the Apple Watch SE uses multiple sensors to precisely monitor heart rate, detect falls, and track physical activity, enhancing the reliability of real-time health insights [9]. Such capabilities demonstrate IoT’s strength in delivering accurate, responsive, and data-driven healthcare solutions. Extra features, such as the Apple Watch’s car crash detection, further exemplify IoT’s potential by facilitating early emergency response through automatic alerts, meeting the rising demand for accessible, cost-effective healthcare [10,11]. Additionally, stationary radar sensors offer non-contact monitoring options for detecting vital signs from a distance, making them particularly useful in-home healthcare settings for elderly or immobile patients. These devices also provide fall detection and remote monitoring capabilities, allowing seniors to live independently while being medically supervised [12].

The benefits of IoT healthcare devices are obvious, but technological advances alone are not enough to make them widely adopted. The use of IoT in healthcare applications depends on user acceptance, which depends on a variety of factors related to perceived risk, trust in the technology, and concerns about data privacy and security [13,14,15]. These issues are particularly important in a sensitive area such as healthcare, where proper handling of personal health information is critical. In addition, risk perception and trust are strongly influenced by demographic characteristics, especially generational differences [16,17].

Indeed, the unified theory of acceptance and use of technology (UTAUT) proposed by Venkatesh et al. [18] provided an effective theoretical framework that might be employed in the investigation and prediction of technology acceptance behavior. The model recognizes four major constructs as influencing users’ behavioral intentions to adopt new technologies: performance expectancy, effort expectancy, social influence, and facilitating conditions. Although the UTAUT model is widely used in various technology acceptance studies, it has a limited capability to capture other factors that have proved important in the context of IoT healthcare devices, such as perceived risk, trust, and privacy concerns. Therefore, extending the UTAUT model to include such factors sheds more light on IoT healthcare adoption, including generational differences [19].

This study extends the UTAUT model by incorporating other variables of attitude, perceived risk, and trust in healthcare contexts. It also develops deeper insight into the moderating influences of generational differences and device type on behavioral intention to adopt IoT devices for home healthcare. It therefore offers useful insights for investigating factors influencing technology acceptance across generational cohorts and thus helps guide development and implementation efforts for IoT healthcare solutions. This study extends the UTAUT model and analyzes generational variations, offering actionable insights to improve public health and enhance quality of life in contexts such as a smart city, which are useful to developers, healthcare professionals, and policymakers.

## 2. Literature Review

### 2.1. Technology Acceptance of Healthcare Devices

The technology acceptance model (TAM), introduced by Davis et al. [20], has been extensively used to explain how users accept and adopt technology, building on the theory of reasoned action (TRA). However, TAM has limitations in addressing external factors, social influences, and the complexity of technology acceptance across different contexts. To address these shortcomings, Venkatesh et al. [18] developed the UTAUT. UTAUT combines elements from eight different models, including TAM, TRA [21], the theory of planned behavior (TPB) [22], the motivational model (MM) [23], combined TAM and TPB (C-TAM-TPB) [24], the model of PC utilization (MPCU) [25], innovation diffusion theory (IDT) [26], and social cognitive theory (SCT) [27], offering a more comprehensive framework for understanding user behavior toward new technologies.

UTAUT identifies four main constructs that significantly influence technology acceptance: performance expectancy, effort expectancy, social influence, and facilitating conditions [18]. Performance expectancy reflects the extent to which a user believes that using technology will enhance their performance, similar to the ‘perceived usefulness’ of TAM. Effort expectancy is related to the perceived ease of technology use, mirroring TAM’s ‘perceived ease of use’. Social influence represents the impact of others’ opinions on an individual’s decision to adopt technology, drawing on the concept of subjective norms from TRA and TPB. Facilitating conditions refer to the user’s perception of the organizational and technical support available for using the technology.

Recent studies applying the UTAUT framework have provided valuable insights into the acceptance of home IoT healthcare devices within smart city environments. For example, Jena [28] expanded UTAUT to explore the adoption of smart services in smart cities, highlighting the importance of performance expectancy, effort expectancy, social influence, and facilitating conditions in driving adoption. Similarly, Popova and Zagulova [29] used UTAUT to investigate how residents use web applications in smart cities, reinforcing the model’s applicability in urban settings. In the healthcare sector, Wang et al. [30] integrated UTAUT with task–technology fit (TTF) to study the acceptance of wearable healthcare devices, showing that the alignment between the technology and users’ tasks is essential for adoption. Kang et al. [31] further supported these findings in their investigation of smart home healthcare services in South Korea, revealing that both UTAUT and TTF constructs significantly influence users‘ behavioral intentions. Additionally, Ben Arfi et al. [17] examined perceived risk and financial costs as barriers to IoT healthcare adoption, emphasizing these as key challenges to overcome. Collectively, these studies demonstrate the robustness of UTAUT in explaining technology acceptance, particularly in the integration of IoT healthcare devices within smart city infrastructures.

### 2.2. Trust

Traditionally, trust existed in interpersonal contexts where individuals relied on others with positive expectations, particularly in situations of uncertainty [32]. However, the rise of digital technologies such as automation, AI, and IoT has further developed the concept of trust. Trust can no longer be conceptualized as strictly being a human-to-human interaction but also as a human–machine interaction [33]. Commonly, trust is closely related to finances, and in the era of e-commerce and digital payments, users need to trust that their data will be rigorously protected [34]. With respect to IoT systems, Fotia et al. provided the idea that users have to trust that these systems will behave as expected, especially in complex scenarios with greater degrees of autonomy [35].

Trust plays a vital role in UTAUT models for healthcare IoT. Trust serves as a mediating factor between the constructs of PE, EE, and SI in the UTAUT model applied to healthcare IoT. It mitigates concerns about privacy, security, and perceived risk, making users more confident in adopting healthcare technologies despite uncertainties [36]. Thus, trust does not just impact behavioral intention (BI) directly but also influences how other factors drive technology adoption, making it a key element in the overall framework. Emphasizing trust not only boosts user engagement but also ensures smoother and more successful implementation of IoT technologies within the healthcare sector [37].

Trust is of greater significance in the context of healthcare, with its sensitive nature of health data and potential risks associated with technological failures [38]. Therefore, trust in H-IoT devices, such as wearable health-monitoring gadgets and remote diagnostic systems, is of utmost concern to guarantee patient and data safety. A malfunction or any security breach in such devices would result in faulty diagnosis or treatment and hence a loss of confidence in healthcare technology [32].

In conclusion, trust has evolved from a simple interpersonal construct to a dynamic and multifaceted concept that is critical in both human and technological interactions. In the context of healthcare IoT, trust is fundamental to the successful adoption and integration of these technologies, relying heavily on security, transparency, and reliability to ensure user confidence [39].

### 2.3. Attitude

Attitude is an individual’s general evaluation or disposition, psychological in nature, toward an object, idea, or action reflecting either favor, disfavor, or neutrality [40]. In UTAUT, attitude plays a crucial role in shaping users’ behavioral intentions. In UTAUT models, attitude is often viewed as an indirect factor that influences the intention to use technology through constructs like performance expectancy, effort expectancy, and social influence [41]. Attitudes help people process information and make decisions. Research has shown that when users hold a positive attitude toward technology, they are more likely to adopt it. Conversely, if their attitude is negative, they may resist its adoption, even if other factors are favorable [42]. Also, stronger attitudes are relatively resistant to change and better predictors of behavior [43].

In addition, attitude is dynamic. It changes over time due to influences from the environment, societal norms, others’ perceptions, or even new personal experiences. Consequently, the association between attitudes and behavioral intentions is quite dynamic [44]. Attitudes can be measured, and one of the early techniques was the Thurstone Attitude Scale, developed by psychologist Louis Thurstone in the 1920s. This provides researchers with the ability to quantify attitudes with scales that integrate the degree to which an individual views a subject positively or negatively [45]. Later, in 1932, Rensis Likert developed the Likert Scale, which quantified attitudes based on respondents’ levels of agreement or disagreement with various statements [46].

In the case of the adoption of home IoT healthcare devices, attitude plays an important role. It reflects an individual’s assessment of the usefulness, safety, and reliability of the technology, whether positive or negative. A more positive perception of these factors tends to lead to higher adoption rates. Therefore, understanding how these factors contribute to users’ attitudes toward IoT technologies is essential for researchers and developers to better predict user behavior and improve the design and implementation of healthcare technologies [47].

### 2.4. Perceived Risk

Perceived risk refers to the psychological discomfort that individuals experience in a state of uncertainty about the occurrence of some specific event, eliciting fear, caution, or a generally negative emotional response. It has received considerable attention in studies on adopting new technologies, mainly for being a critical factor influencing user behavior, with a perfect measurement scale for reference [47,48]. Perceived risk (PR), as an important variable, expands the UTAUT model by impacting key constructs like behavioral intention [36]. Perceived risk is regarded as the loss that may occur while trying to deduce the desired result from electronic services. It encompasses risks to performance, finances, time, information privacy, and psychological stressors [49].

Though IoT devices can enhance efficiency and effectiveness in the delivery of health services, high levels of perceived risk lower user trust and negatively affect user’s intention to adopt the technologies. Customers may view this as a threat to their privacy [17]. Additionally, to subscribe to any e-health service, all the providers must enter sensitive financial and personal details, which may also lead to customers perceiving a kind of risk [50]. Despite years of research into IoT adoption in healthcare devices, gaps still need to be filled, especially in aspects such as privacy and data protection in general, and trust in connected healthcare devices. More precisely, perceived risk and trust have been considered critical factors for changing user behaviors and the adoption of IoT in healthcare systems [51]. Research has shown that reducing perceived risk is an effective way to foster trust, increase behavioral intention, and promote widespread adoption of IoT healthcare technologies [15,17]. 

Therefore, the following hypotheses are proposed in this study:

**H1.**  *Performance expectancy positively impacts behavioral intention in adopting IoT healthcare devices*.

**H2.**  *Effort expectancy positively impacts behavioral intention in adopting IoT healthcare devices*.

**H3.**  *Social influence positively impacts behavioral intention in adopting IoT healthcare devices*.

**H4.**  *Facilitating conditions positively impact behavioral intention in adopting IoT healthcare devices*.

**H5.**  *Perceived risk negatively impacts behavioral intention to adopt IoT healthcare devices*.

**H6.**  *Performance expectancy positively impacts trust in IoT healthcare devices*.

**H7.**  *Effort expectancy positively impacts trust in IoT healthcare devices*.

**H8.**  *Social influence positively impacts trust in IoT healthcare devices*.

**H9.**  *Facilitating conditions positively impact trust in IoT healthcare devices*.

**H10.**  *Perceived risk negatively impacts trust in IoT healthcare devices*.

**H11.**  *Performance expectancy positively impacts attitude toward using IoT healthcare devices*.

**H12.**  *Effort expectancy positively impacts attitude toward using IoT healthcare devices*.

**H13.**  *Social influence positively impacts attitude toward using IoT healthcare devices*.

**H14.**  *Facilitating conditions positively impact attitude toward using IoT healthcare devices*.

**H15.**  *Perceived risk negatively impacts attitude toward using IoT healthcare devices*.

**H16.**  *Trust positively impacts behavioral intention to adopt IoT healthcare devices*.

**H17.**  *Attitude positively impacts behavioral intention to adopt IoT healthcare devices*.

### 2.5. The Role of Generation in Technology Acceptance

There are generational differences that are critical in shaping technology adoption behaviors. Generation XY, born between 1966 and 1994, has been described as “digital immigrants” since they grew up in the transition from analog to digital media [52,53]. They can be more cautious with new technologies, particularly those associated with privacy and data security risks. They are likely to evaluate the potential benefits of IoT healthcare devices against possible drawbacks, such as data breaches, loss of control over personal information, and the reliability of the devices themselves. Trust in the technology and its providers plays a significant role in determining whether Generation XY will adopt IoT healthcare solutions [54,55].

In contrast, Generation Z, born after 1994 and commonly referred to as “digital natives”, has grown up in a world where digital technology is ubiquitous. They are generally more comfortable integrating technology into various aspects of their lives, including healthcare [56,57]. This generation values convenience, seamless integration with other digital platforms, and user-friendly interfaces that allow for easy management of personal health data. Their acceptance of IoT healthcare devices is likely to be driven by familiarity with digital technologies and expectations of instant access to information and continuous connectivity. Therefore, understanding these generational differences is crucial for the successful implementation of IoT healthcare solutions, as strategies for promoting adoption must address the specific needs and concerns of each cohort [58,59]. Therefore, the following hypotheses are proposed:

**H18a.**  *Generation moderates the relationship between trust and behavioral intention*.

**H18b.**  *Generation moderates the relationship between attitude and behavioral intention*.

### 2.6. The Role of Device Types in Technology Acceptance

The moderating role of device type in technology acceptance is a critical aspect of understanding user behavior. Devices vary in usability, mobility, and functionality, influencing how users perceive and interact with technology. For example, research on mobile shopping applications has shown that device type significantly affects perceived usefulness, perceived enjoyment, and satisfaction [60].

In healthcare, the moderating effect of device type becomes even more pronounced, especially with wearables and smart healthcare devices. For instance, users’ trust and perceptions of risk are significantly influenced by the type of device used. As wearable devices integrate with existing devices and platforms, they may inspire higher trust from users since they are perceived as extensions of their personal technology ecosystem [15]. On the other hand, more complex radar sensors could give people a sense of intrusion or difficulty in understanding them, thereby elevating the perceived risk level [38].

A significant impact of device type on perceived usefulness and satisfaction has been found in research. For instance, Natarajan et al. [60] found that mobile devices’ portability and ease of use enhance perceived enjoyment and satisfaction in mobile shopping applications, suggesting that the context of use plays a critical role in acceptance. In healthcare, wearables and smart devices offer unique benefits and limitations. As a result of the ability to monitor and collect data continuously, wearable devices have become a highly valuable tool for managing one’s health [8]. In addition to the accuracy of data, user comfort can also present challenges, which negatively impact acceptance.

The characteristics of devices also moderate the relationship between key UTAUT constructs and the degree of technology acceptance. For example, devices that are more familiar to users, such as smartphones, may have higher performance expectancy and effort expectancy due to their widespread use and intuitive interfaces [31]. Comparatively, advanced medical sensors may require greater facilitating conditions, such as extensive user training and support, to achieve similar acceptance levels.

Based on these findings, the following hypotheses are proposed:

**H19a.**  *Device type moderates the relationship between trust and behavioral intention*.

**H19b.**  *Device type moderates the relationship between attitude and behavioral intention*.

## 3. Methodology

**Figure 1:**
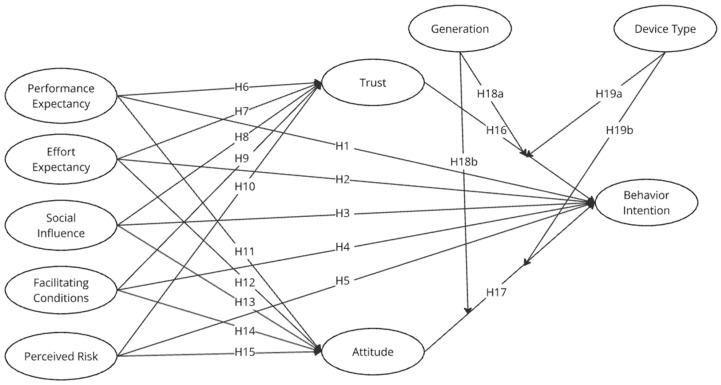
Research model.

Based on previous studies and the proposed hypotheses, the research model illustrated in Figure 1 is designed to examine the factors influencing the technology acceptance of healthcare devices.

**Figure 2:**
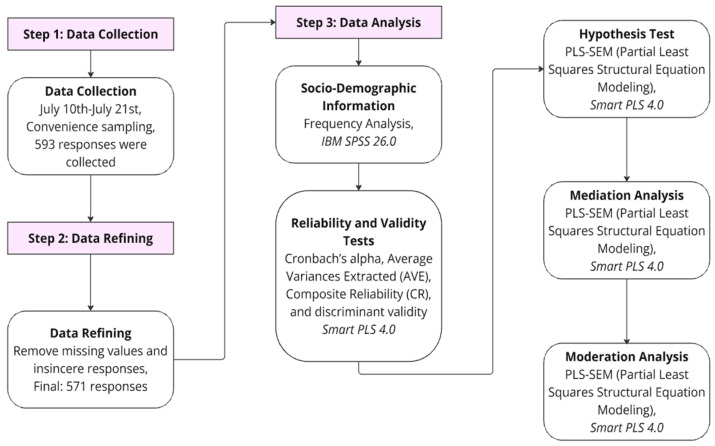
Research flow.

This study followed a structured research design comprising four key stages: data collection, data refining, and data analysis, as illustrated in Figure 2. Data collection took place from June 10th to July 21st through an online survey distributed through Google Forms, using convenience sampling. The survey focused on two specific types of home IoT healthcare devices: a 60 GHz radar sensor for heartbeat and respiration monitoring and a wearable device (Apple Watch SE) with features for health and safety monitoring, including heart rate tracking and ECG capabilities. A total of 593 responses were initially collected for each device type, resulting in 1186 responses. After implementing a data refining process to ensure data quality, responses with missing information or insincere answers were excluded. This resulted in 579 valid responses for each device type, providing a final dataset of 1158 responses for hypothesis testing, moderation, and mediation analysis.

The refined dataset underwent the following analysis: First, the socio-demographic information of respondents was generated using SPSS 26.0. Then, the reliability and validity of the constructs were tested using SmartPLS 4.0 to validate the measurement model. Hypotheses were tested through SmartPLS 4.0 to examine the relationships proposed in the research model. Additionally, the mediation effects of attitude and trust between the independent variables and behavioral intention were analyzed using SmartPLS, followed by an examination of the moderation effects of generation and device type.

## 4. Results

### 4.1. Demographic Information

The demographic information in Table 1 shows a near-equal gender split, with 49.6% of respondents being male and 50.4% female. The largest age group consists of individuals in their 20s (52.4%), followed by those in their 30s (32.6%). A smaller proportion are either younger than 20 (2.9%) or older than 40 (12.1%). In terms of nationality, Indonesians make up the majority (52.2%), while Chinese (32.8%) and Vietnamese (11.7%) participants form significant portions as well. Regarding educational background, most respondents hold a bachelor’s degree (60.8%), with fewer having postgraduate degrees (15.7%), attending a 3-year college (14.9%), or finishing high school or below (8.6%).

Income levels show that almost half of the respondents (48.1%) earn between USD 1000 and 1999 monthly, while 22.5% make less than USD 1000, another 22.5% earn between USD 2000 and 2999, and 6.9% report earning over USD 3000. Marital status reveals that 43.2% are married, 39.5% are single, and 17.3% are divorced. As for household size, the majority live in households with three people (27.0%) or more than four people (27.6%). Additionally, 24.7% live in two-person households, and 20.7% reside alone.

### 4.2. Reliability and Validity Test

This study evaluated the validity and reliability of reflective measurement models using PLS-SEM in accordance with the guidelines provided by Hair et al. [61], as shown in Table 2. Both indicator-level and construct-level reflective measurement models were evaluated, and the measures’ reliability was examined.

The structures fulfill the given criteria, indicating that each item correctly reflects its corresponding construct. Hulland [62] states that factor loadings should be greater than 0.7, and all of the items in each of the constructs satisfy this requirement, meaning that every item is a powerful indicator of the construct for which it is intended. Bagozzi and Yi [63] state that composite reliability (CR) should be above 0.7. The CR values for each construct range between 0.777 and 0.831, indicating that the items are internally consistent. Furthermore, the average variance extracted (AVE) values, which should be greater than 0.5 as indicated by Bagozzi and Yi [63], range from 0.656 to 0.747, showing that the constructs account for a significant amount of variance in their items. Furthermore, all Cronbach’s alpha values are above 0.7 [64], indicating the scales’ reliability. All of these results support the validity and reliability of the constructs and provide a strong foundation for additional research.

The next step involved assessing discriminant validity, which evaluates how distinct a construct is from others within the structural model. In this study, the Fornell–Larcker criterion was applied to determine discriminant validity. According to Fornell and Larcker [65], it is important to ensure that the shared variance between constructs is lower than their respective average variance extracted (AVE), indicating that each construct effectively captures its own unique variance. As shown in Table 3, all constructs meet this requirement, confirming their discriminant validity.

### 4.3. Hypotheses Testing Results

The results of the hypothesis testing provide detailed insights into the factors influencing user attitudes (Att), trust (T), and behavioral intentions (BI) toward adopting healthcare devices. As shown in Table 4, hypotheses H1 through H5 demonstrate that performance expectancy (PE), effort expectancy (EE), social influence (SI), facilitating conditions (FC), and perceived risk (PR) significantly influence user attitudes. In the analysis, the perceived risk variable was reverse-coded to ensure consistency in interpretation, where higher values indicated lower levels of perceived risk. PE (H1, *β* = 0.194, *p* < 0.001), EE (H2, *β* = 0.189, *p* < 0.001), SI (H3, *β* = 0.137, *p* < 0.001), FC (H4, *β* = 0.228, *p* < 0.001), and PR (H5, *β* = 0.178, *p* < 0.001) all have positive effects, indicating that users develop favorable attitudes toward healthcare devices when they find them useful, easy to use, and supported by their social environment and available resources, while lower perceived risks also enhance user attitudes.

Trust is also significantly impacted by these variables. Hypotheses H6 through H10 show that PE (H6, *β* = 0.220, *p* < 0.001), EE (H7, *β* = 0.164, *p* < 0.001), SI (H8, *β* = 0.215, *p* < 0.001), FC (H9, *β* = 0.232, *p* < 0.001), and PR (H10, *β* = 0.141, *p* < 0.001) all positively influence trust, indicating that these factors not only shape attitudes but also build user trust in healthcare devices. For behavioral intention, however, the effects of PE (H11, *β* = 0.034, *p* = 0.335) and EE (H12, *β* = 0.050, *p* = 0.159) are not significant. In contrast, SI (13, *β* = 0.194, *p* < 0.001), FC (H14, *β* = 0.129, *p* < 0.001), PR (H15, *β* = 0.169, *p* < 0.001), attitude (H16, *β* = 0.312, *p* < 0.001), and trust (H17, *β* = 0.100, *p* = 0.042) all significantly affect BI. Attitude emerges as the strongest predictor of behavioral intention, showing that favorable attitudes, combined with social influence, facilitating conditions, and trust, drive the intention to adopt healthcare devices.

### 4.4. Mediation Effect Results

As shown in Table 5, the results of the mediation analysis revealed both full and partial mediation effects, highlighting the roles of attitude and trust in influencing the relationship between independent variables and behavioral intention. For the paths involving performance expectancy and effort expectancy, attitude fully mediates their effects on BI. Specifically, PE (indirect effect *β* = 0.060, *p* < 0.001) and EE (indirect effect *β* = 0.059, *p* < 0.001) influence BI only through their impact on attitude, as the direct effects on BI are not significant (*p* = 0.335 and *p* = 0.159, respectively). For social influence, facilitating conditions, and perceived risk, partial mediation is observed. While these factors directly influence BI (*p* < 0.001), they also have significant indirect effects through attitude, as indicated by indirect effect coefficients for SI (*β* = 0.043, *p* = 0.003), FC (*β* = 0.071, *p* < 0.001), and PR (*β* = 0.055, *p* < 0.001).

In contrast, no significant mediation effect is found when trust serves as the mediator for the relationship between PE, EE, SI, FC, PR, and BI. Although trust contributes to explaining BI, the direct effects of these factors on BI remain significant, indicating that trust does not fully mediate the relationship. For instance, PE (indirect effect *β* = 0.022, *p* = 0.054), EE (indirect effect *β* = 0.016, *p* = 0.063), and the other variables show no significant mediation effect through trust, as the confidence intervals suggest limited mediation.

### 4.5. Moderation Effect Results

The results of the moderation analysis indicate that the effect of generation plays a significant role in moderating the relationships of both attitude and trust with behavioral intention, as shown in Table 6. Specifically, the interaction term for Generation x Att -> BI is significant (*β* = −0.297, *t* = 4.984, *p* < 0.001), suggesting that the impact of attitude on behavioral intention is moderated by generation, with the relationship becoming weaker for certain age groups. Similarly, the interaction for Generation x T -> BI is also significant (*β* = 0.285, *t* = 4.609, *p* < 0.001), indicating that the influence of trust on behavioral intention varies across generations, with the effect being stronger for some generational cohorts.

In contrast, the moderation effect of device type on the relationship between attitude and behavioral intention is not significant (*β* = 0.052, *t* = 0.803, *p* = 0.422), meaning that the type of device (radar sensor or wearable) does not significantly alter the impact of attitude on behavioral intention. However, device type does have a significant moderating effect on the relationship between trust and behavioral intention (*β* = −0.138, *t* = 2.099, *p* = 0.036), suggesting that trust influences behavioral intention differently depending on the device type, with a weaker relationship for one of the devices. These findings highlight the importance of considering both generational differences and device type when examining how attitude and trust influence the adoption of healthcare devices.

## 5. Discussion and Implications

### 5.1. Discussion

The results of this study provide insights into how various factors influence user attitudes, trust, and behavioral intentions toward adopting healthcare devices. Performance expectancy, effort expectancy, social influence, facilitating conditions, and perceived risk were all found to have significant effects on user attitudes and trust, which in turn shape behavioral intention. Specifically, while PE and EE are traditionally considered direct drivers of BI, the findings suggest that their effects on BI are fully mediated by attitude. This indicates that even if users perceive the devices to be useful or easy to use, these perceptions must first result in positive attitudes before they impact the intention to adopt. These findings add nuance to the existing understanding of technology acceptance, particularly in the healthcare context.

Moreover, the mediation analysis reveals that attitude plays a critical role in the adoption process, fully mediating the relationships between PE, EE, and BI. This means that user perceptions of a healthcare device’s performance and effort requirements must be translated into favorable attitudes before influencing their intention to adopt. On the other hand, SI, FC, and PR exhibit partial mediation through attitude, suggesting that while they directly influence BI, they also have indirect effects by shaping user attitudes. Trust, while significantly influenced by these factors, does not fully mediate their effects on BI. This nuanced interplay between attitude, trust, and BI suggests that fostering a positive user mindset is crucial for promoting the adoption of healthcare devices.

The moderation analysis further underscores the importance of considering individual differences, particularly generational and device-type factors. The effect of generation as a moderator shows that younger and older generations differ in how attitude and trust influence their intention to adopt healthcare devices. Additionally, the moderating effect of device type reveals that trust influences BI differently depending on whether the device is a wearable or a radar sensor. This suggests that strategies to enhance trust may need to be tailored to the specific type of healthcare device, further highlighting the importance of context in technology adoption research.

### 5.2. Theoretical Implications

This research makes a significant contribution to the theoretical understanding of technology acceptance in the healthcare area by extending the UTAUT model to integrate constructs such as attitude, trust, and perceived risk. By demonstrating how users develop positive attitudes and trust in healthcare technologies, this study reinforces the relevance of UTAUT and aligns with the findings of previous research [14,17,18].

This study also highlighted the role of attitude, as suggested in previous studies on technology acceptance [65,66]. This insight provides a deeper understanding of how user perceptions are processed and underscores the importance of positive attitudes in driving the adoption of healthcare devices. By identifying this mediation effect, this study enriches the UTAUT model and suggests that future models should account for the role of attitude in the adoption of complex technologies such as healthcare devices.

However, trust does not fully mediate the relationships between the UTAUT constructs and BI. While trust plays a role in explaining BI, its lack of full mediation suggests that other factors, such as attitude, have a more substantial direct impact on behavioral intention. This lack of full mediation by trust can be explained through several theoretical perspectives and previous literature. Trust is often seen as a critical factor in technology adoption, particularly in contexts involving high uncertainty and perceived risk, such as healthcare. However, the role of trust can vary significantly depending on the specific context and the nature of the technology being adopted. Furthermore, when users make a decision to adopt healthcare technology, they take into account a variety of factors. The trust factor is one of these factors.

There is also evidence that generational differences can affect how trust mediates the relationship between UTAUT constructs and BI. As younger users integrate technology into their daily lives, they may rely more on their attitude and perceived ease of use than just trust alone, whereas older users might place a greater emphasis on trust [67].

Trust can influence BI directly or indirectly by enhancing the perceived credibility and reliability of the technology. In some studies, trust has been shown to directly impact BI without fully mediating other antecedents [68]. Accordingly, trust is not necessarily essential for adoption, but rather plays a nuanced and context-dependent role. Moreover, in the context of healthcare devices, users have the tendency to form attitudes based on the ease of use, usefulness, and social influence of the device, which directly affect their behavior [69]. Positive attitudes would reduce the direct influence of trust.

Moreover, the moderation effects of generation and device type highlight the importance of contextual factors in technology acceptance research, in alignment with previous studies [70,71]. Generation-based moderation shows that younger and older users differ in how their attitudes and trust influence their behavioral intentions, suggesting that generational differences are critical in understanding healthcare technology adoption. On the other hand, the finding that device type moderates the relationship between trust and behavioral intention but not between attitude and behavioral intention implies that the nature of the device can affect trust differently. This finding opens new theoretical avenues for exploring how specific technological features, such as the form factor or functionality of healthcare devices, impact trust and ultimately user adoption.

### 5.3. Practical Implications

The findings of this study offer several practical implications for healthcare device manufacturers, policymakers, and healthcare providers. First, the critical role of attitude in influencing behavioral intention suggests that fostering positive user attitudes should be a top priority for promoting the adoption of healthcare devices. Manufacturers should focus on designing devices that are not only functional but also easy to use, intuitive, and aligned with user expectations. By ensuring that users have positive initial experiences with the technology, companies can cultivate favorable attitudes that are more likely to translate into adoption. Additionally, communicating the clear benefits of the devices, both in terms of health outcomes and convenience, can further enhance user attitudes.

Generational differences also have important implications for marketing and outreach strategies. Since younger users are more influenced by attitudes, promotional efforts targeted at this group should emphasize the ease of use and performance benefits of healthcare devices. For older generations, where trust plays a more prominent role, manufacturers and healthcare providers should focus on building trust through reliable performance, data security, and long-term support. Providing detailed information about the device’s privacy protections and reliability can help alleviate concerns among older users, increasing their likelihood of adopting the technology. Additionally, the role of social influence suggests that endorsements from trusted healthcare professionals or testimonials from satisfied users can further bolster adoption, especially among older generations who may be more cautious.

Device-specific strategies are also necessary, as the moderation effect of device type reveals that users perceive different types of healthcare devices in unique ways. For more complex or unfamiliar technologies, such as radar sensor systems, efforts to build trust may need to be more pronounced [72]. This could involve providing extensive user education, offering hands-on demonstrations, or ensuring robust customer support. On the other hand, wearable devices, which may be more familiar to users, might benefit from efforts that focus on improving user experience and convenience. Additionally, improving facilitating conditions, such as technical support and user-friendly interfaces, can further drive adoption by reducing barriers and ensuring that users feel confident in using the devices [73]. Reducing perceived risk, especially in terms of data privacy and device reliability, should be a key focus across all device types to increase user trust and facilitate broader adoption.

### 5.4. Limitations and Future Studies

Despite the valuable insights gained from this study, several limitations should be acknowledged. First, the use of a convenience sampling method may limit the generalizability of the findings, as the sample may not fully represent the broader population. Additionally, the study focused on healthcare devices, specifically wearable devices and radar sensors, which may not capture the full spectrum of user experiences and technology acceptance across different types of devices. This restricts the ability to generalize the results to other healthcare technologies that might evoke different user responses.

Therefore, future research should aim to address these limitations by employing random sampling methods to enhance the representativeness and reliability of the findings. Moreover, exploring a broader range of healthcare devices would provide a more comprehensive understanding of user behavior and acceptance across various technologies. Further investigation into the role of trust as a mediator is also necessary, as it did not have a significant impact in this study. Future studies could explore alternative contexts or variables where trust may play a more prominent role in influencing behavioral intention, potentially uncovering more insights into its impact on technology adoption.

## Figures and Tables

**Table 1:** Demographic information (*n* = 579).

	Variables	Frequency	Percent (%)
Gender	Male	287	49.6
	Female	292	50.4
Generation	Under 20	17	2.9
	20s	303	52.4
	30s	189	32.6
	Over 40	70	12.1
Nationality	Korean	17	2.9
	Indonesian	302	52.2
	Vietnamese	68	11.7
	Chinese	190	32.8
	Others	2	0.4
Education Level	High school graduate and below	50	8.6
	3-year college	86	14.9
	Bachelor’s degree	352	60.8
	Postgraduate	91	15.7
Monthly Income	Less than USD 1000	130	22.5
	USD 1000~1999	279	48.1
	USD 2000~2999	130	22.5
	Over USD 3000	40	6.9
Marital Status	Not married	229	39.5
	Married	250	43.2
	Divorced	100	17.3
Household #	1 (living alone)	120	20.7
	2	143	24.7
	3	156	27.0
	More than 4	160	27.6

**Table 2:** Reliability and validity test.

Constructs	Items	Factor Loading	CR	AVE	Cronbach’s Alpha
Performance Expectancy	PE1	0.882	0.821	0.732	0.816
	PE2	0.818			
	PE3	0.865			
Effort Expectancy	EE1	0.897	0.831	0.747	0.830
	EE2	0.856			
	EE3	0.838			
Social Influence	SI1	0.862	0.793	0.704	0.790
	SI2	0.808			
	SI3	0.847			
Facilitating Conditions	FC1	0.872	0.792	0.704	0.789
	FC2	0.817			
	FC3	0.826			
Perceived Risk	CI1	0.810	0.777	0.688	0.774
	CI2	0.835			
	CI3	0.843			
Trust	T1	0.868	0.827	0.656	0.824
	T2	0.778			
	T3	0.783			
	T4	0.806			
Attitude	Att1	0.884	0.782	0.695	0.779
	Att2	0.765			
	Att3	0.848			
Behavioral Intention	BI1	0.855	0.829	0.657	0.825
	BI2	0.760			
	BI3	0.789			
	BI4	0.835			

Note. CR: composite reliability, AVE: average variance extracted.

**Table 3:** Discriminant validity test (Fornell–Larcker criterion).

	Att	BI	EE	FC	PE	PR	SI	T
Att	**0.834**							
BI	0.791	**0.811**						
EE	0.733	0.735	**0.864**					
FC	0.753	0.779	0.771	**0.839**				
PE	0.735	0.743	0.769	0.753	**0.855**			
PR	0.704	0.764	0.678	0.719	0.680	**0.829**		
SI	0.725	0.793	0.716	0.765	0.749	0.744	**0.839**	
T	0.795	0.799	0.761	0.789	0.777	0.728	0.779	**0.810**

Note. Att: attitude; BI: behavioral intention; EE: effort expectancy; FC: facilitating conditions; PE: performance expectancy; PR: perceived risk; SI: social influence; T: trust. Perceived risk (PR) is a reversed variable. Values in bold represent the square root of the variance extracted (AVE) and the values outside the diagonal represent the correlations between the constructs.

**Table 4:** Summary of hypotheses testing results.

Path		Path Coefficient (*β*)	Standard Deviation	*t*-Value	*p*-Value	Result
H1	PE -> BI	0.034	0.036	0.964	0.335	Not Supported
H2	EE -> BI	0.050	0.036	1.408	0.159	Not Supported
H3	SI -> BI	0.194	0.045	4.350	<0.001	Supported
H4	FC -> BI	0.129	0.033	3.848	<0.001	Supported
H5	PR -> BI	0.169	0.038	4.450	<0.001	Supported
H6	PE -> T	0.220	0.037	5.971	<0.001	Supported
H7	EE -> T	0.164	0.037	4.466	<0.001	Supported
H8	SI -> T	0.215	0.038	5.613	<0.001	Supported
H9	FC -> T	0.232	0.038	6.029	<0.001	Supported
H10	PR -> T	0.141	0.035	3.969	<0.001	Supported
H11	PE -> Att	0.194	0.041	4.732	<0.001	Supported
H12	EE -> Att	0.189	0.038	4.982	<0.001	Supported
H13	SI -> Att	0.137	0.040	3.439	<0.001	Supported
H14	FC -> Att	0.228	0.037	6.211	<0.001	Supported
H15	PR -> Att	0.178	0.035	5.115	<0.001	Supported
H16	Att -> BI	0.312	0.049	6.296	<0.001	Supported
H17	T -> BI	0.100	0.049	2.036	0.042	Supported

Note. PE: performance expectancy; EE: effort expectancy; SI: social influence; FC: facilitating conditions; PR: perceived risk; T: trust; Att: attitude; BI: behavioral intention. Perceived risk (PR) is a reversed variable. Higher values indicate lower perceived risk.

**Table 5:** Results of mediation analysis.

Paths	Total Effect	Direct Effect	Indirect Effect			95% CI		Mediation Effect
	*β*	*β*	*p*-Value	*β*	*p*-Value	Lower	Upper	
PE -> Att -> BI	0.083	0.034	0.335	0.060	<0.001	0.031	0.095	Full mediation effect
EE -> Att -> BI	0.075	0.050	0.159	0.059	<0.001	0.034	0.088	Full mediation effect
SI -> Att -> BI	0.064	0.194	<0.001	0.043	0.003	0.018	0.073	Partial mediation effect
FC -> Att -> BI	0.094	0.129	<0.001	0.071	<0.001	0.041	0.105	Partial mediation effect
PR -> Att -> BI	0.070	0.169	<0.001	0.055	<0.001	0.029	0.085	Partial mediation effect
PE -> T -> BI	0.083	0.034	0.335	0.022	0.054	0.001	0.046	No mediation effect
EE -> T -> BI	0.094	0.050	0.159	0.016	0.063	0.001	0.036	No mediation effect
SI -> T -> BI	0.064	0.194	<0.001	0.022	0.063	0.001	0.046	No mediation effect
FC -> T -> BI	0.094	0.129	<0.001	0.023	0.055	0.001	0.048	No mediation effect
PR -> T -> BI	0.070	0.169	<0.001	0.014	0.089	0.001	0.033	No mediation effect

Note. PE: performance expectancy; EE: effort expectancy; SI: social influence; FC: facilitating conditions; PR: perceived risk; T: trust; Att: attitude; BI: behavioral intention. Perceived risk (PR) is a reversed variable. Higher values indicate lower perceived risk.

**Table 6:** Results of moderation analysis.

Paths	Standard Deviation	Standard Deviation	*t*-Value	*p*-Value	Result
Generation x Att -> BI	−0.297	0.059	4.984	<0.001	Supported
Generation x T -> BI	0.285	0.062	4.609	<0.001	Supported
Device Type x Att -> BI	0.052	0.065	0.803	0.422	Not Supported
Device Type x T -> BI	−0.138	0.066	2.099	0.036	Supported

Note. T: trust; Att: attitude; BI: behavioral intention.

## Data Availability

Data presented in this study are available on reasonable request from the corresponding author.
